# Addressing Peroxisome Proliferator-Activated Receptor-gamma in 3-Nitropropionic Acid-Induced Striatal Neurotoxicity in Rats

**DOI:** 10.1007/s12035-022-02856-w

**Published:** 2022-05-12

**Authors:** Riham M. Mansour, Nesrine S. El Sayed, Maha A. E. Ahmed, Ayman E. El-Sahar

**Affiliations:** 1 Department of Pharmacology and Toxicology, College of Pharmaceutical Sciences and Drug Manufacturing, Misr University for Science and Technology (MUST), 6Th of October City, Giza Egypt; 2 Department of Pharmacology and Toxicology, Faculty of Pharmacy, Cairo University, Kasr El-Aini Street, Cairo, Egypt

**Keywords:** Neurotoxicity, Striatum, 3-nitropropionic acid, Peroxisome proliferator-activated receptor gamma (PPARγ)

## Abstract

Telmisartan (TEL) is an angiotensin II type 1 receptor blocker and a partial activator of peroxisome proliferator-activated receptor-gamma (PPARγ), which regulates inflammatory and apoptotic pathways. Increasing evidence has demonstrated the PPARγ agonistic property of TEL in several brain disorders. This study aims to explore the neuroprotective impact of TEL in 3-nitropropionic acid (3-NP)-induced neurotoxicity in rats. The PPARγ effect of TEL was affirmed by using the PPARγ agonist pioglitazone (PIO), and the antagonist GW9662. 3-NP led to a significant reduction in body weight alongside motor and cognitive functioning. The striata of the 3-NP-treated rats showed energy-deficit, microglia-mediated inflammatory reactions, apoptotic damage as well as histopathological lesions. PIO and TEL improved motor and cognitive perturbations induced by 3-NP, as confirmed by striatal histopathological examination, energy restoration, and neuronal preservation. Both drugs improved mitochondrial biogenesis evidenced by elevated mRNA expression of PPARγ, PGC-1α, and TFAM, alongside increased striatal ATP and SDH. The mitochondrial effect of TEL was beyond PPARγ activation. As well, their anti-inflammatory effect was attributed to suppression of microglial activation, and protein expression of pS536 p65 NF-κB with marked attenuation of striatal inflammatory mediator's release. Anti-inflammatory cytokine IL-10 expression was concurrently increased. TEL effectively participated in neuronal survival as it promoted phosphorylation of Akt/GSK-3β, further increased Bcl-2 expression, and inhibited cleavage of caspase-3. Interestingly, co-treatment with GW9662 partially revoked the beneficial effects of TEL. These findings recommend that TEL improves motor and cognitive performance, while reducing neuronal inflammation and apoptosis in 3-NP-induced neurotoxicity via a PPARγ-dependent mechanism.

**Graphical Abstract:**

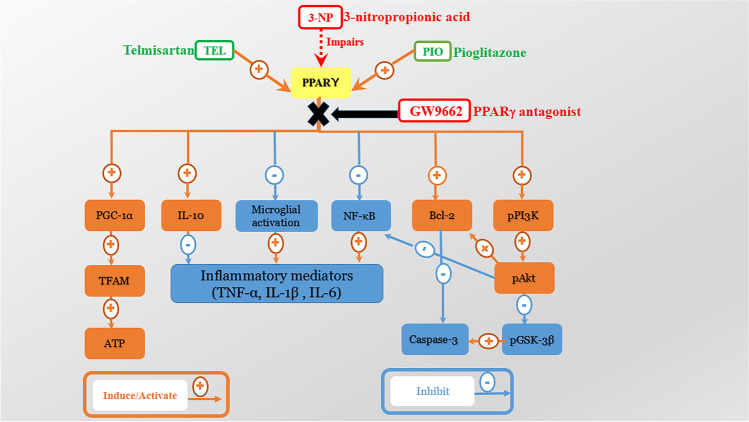

## Introduction

Disorders of movement, cognition, and behavior constitute the major observed abnormalities in neurodegenerative diseases, including Huntington’s disease (HD). This dominant inheritable disorder has been associated with the expression of mutated huntingtin (mHTT) protein, which actuates a set of deleterious events terminating in selective loss of striatal neurons in HD [1].

The toxic animal model of 3-nitropropionic acid (3-NP) has been widely used to replicate the neurobehavioral and biochemical changes that are reminiscent of those occurring in HD [2]. Research has shown that 3-NP can easily pass the blood–brain barrier (BBB) and irreversibly inhibit succinate dehydrogenase (SDH, mitochondrial complex II), thus blocking energy production. Further, 3-NP promotes cortical and striatal neuronal loss, microglial activation, pro-inflammatory cytokine release, ATP depletion, and neuronal apoptosis [3, 4].

Neuroinflammation, bioenergetics failure, and transcriptional dysregulation are the pivotal events leading to neurodegeneration occurring in HD. Interestingly, the nuclear receptor, peroxisome proliferator-activated receptor-gamma (PPARγ) has been recognized as a therapeutic target for various neurodegenerative disorders such as Alzheimer's (AD), Parkinson's (PD), and HD [5]. Multiple studies have demonstrated that PPARγ agonism, as well as PPARγ-dependent neuroprotection, could attenuate the inflammatory and apoptotic responses as well as promote mitochondrial biogenesis [6–8]. PPARγ is a ligand-activated transcription factor that regulates multiple pathways involved in carbohydrate and lipid metabolism [9].

Most neuroinflammatory diseases have been accompanied by activation of microglia, which secrete a panel of pro-inflammatory mediators such as tumor necrosis factor α (TNF-α), interleukin (IL)-1β, IL-6, nitric oxide, and cyclooxygenase 2, that propagate neuronal injury [10]. Lots of evidence showed that PPARγ could blunt microglial activation, thus controlling the inflammatory milieu in several models of neurodegenerative diseases [11]. In addition, PPARγ activation can regulate inflammatory pathways by transrepression of the transcription factor nuclear factor kappa B (NF-κB) [12], with the consequent reduction in neuroinflammatory mediators. Moreover, PPARγ exerts neuroprotective effects by inducing neuronal differentiation and neurite outgrowth [13].

It has been documented that the transcription dysregulation of PPARγ and its downstream target PPARγ co-activator-1 alpha (PGC-1α) contributes to the mitochondrial impairment in HD [14, 15]. PGC-1α is a key regulator in the circuitry of transcriptional machinery terminating in enhanced mitochondrial energy production [16]. In regard, the role of PPARγ in HD has been explored in cell models [17, 18], chemically induced [19, 20], and transgenic HD models [14, 15, 18].

Evidence has shown the involvement of PPARγ in survival pathways, such as the phosphoinositide 3-kinase/protein kinase B (PI3K/Akt), which blocks apoptosis progression and maintains neuronal vitality [21]. In addition, Akt can phosphorylate GSK-3β at serine9 and inactivate this enzyme, thus preventing GSK-3β from initiating an apoptotic pathway [22]. Lately, GSK-3 inhibition is considered a druggable target to ameliorate HD pathogenesis as it reduced the striatal aggregates of mHTT and enhanced motor and coordination skills [23].

The angiotensin II type 1 receptor blockers (ARBs) are widely employed to control neurodegenerative disorders, not only for their AT1-blocking functions, but also due to their PPARγ partial agonistic activity [24], therapy controlling neuroinflammation and apoptotic response. Of interest, telmisartan is the ARB with the greatest PPARγ agonist activity [25]. Recently, several studies have shown that telmisartan exerts multiple neuroprotective effects through PPARγ activation in vitro [26–29] and in vivo [30–35]. However, it keeps unknown whether PPARγ activation contributed to the neuroprotective effects of telmisartan in the model of HD.

This work aims to explore whether telmisartan provides neuroprotection against striatal degeneration, energy deficits, and neuroinflammation in a rat 3-NP model of HD, and reveal PPARγ-related signaling.

## Material and Methods

### Animals

Male rats of Wistar strain (200–250 g) were obtained from the National Scientific Research Centre (Giza, Egypt). The rats were housed in clean plastic cages on a 12-h light / dark cycle, 25±2°C temperature, 60±10 % humidity with free access to food and water. Animals were acclimatized to the laboratory condition one week before experimentation. The study protocols follow the Guide for the Use and Care of Laboratory Animals published by the US National Institutes of Health (NIH Publication No. 85-23, revised 2011) and was conducted according to the ethical procedures and policies approved by the Ethics Committee of Faculty of Pharmacy, Cairo University (Permit Number: PT 2769).

### Drugs and Reagents

3-NP, dimethyl sulfoxide (DMSO), and carboxymethyl cellulose (CMC) were procured from Sigma-Aldrich, St. Louis, MD, USA. Telmisartan (Micardis^®^) was purchased from Boehringer Ingelheim, Germany. Pioglitazone (Actos^®^) was purchased from Takeda Pharmaceuticals Ltd. The PPARγ antagonist, GW9662, was purchased from MedChemExpress, NJ, USA. All other chemicals and reagents used in this study were of analytical grade.

### Experimental Procedure

**Fig. 1:**
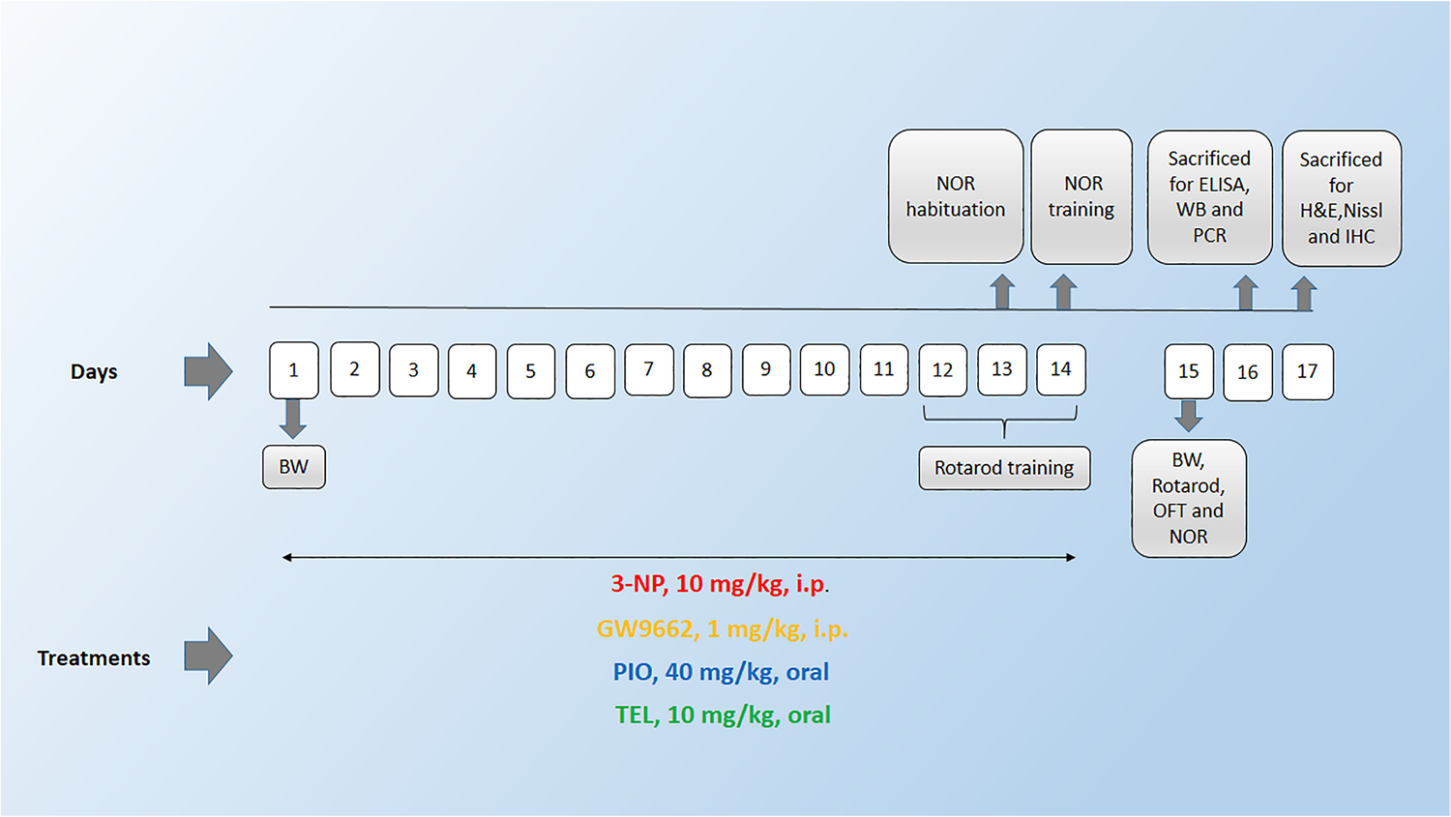
Schedule of the experimental study. (PIO: pioglitazone; TEL: telmisartan; 3-NP: 3-nitropropionic acid; IHC: immunohistochemistry; WB: Western blot; BW: body weight; OFT: open field test; NOR: novel object recognition test)

Rats were randomly assigned into six groups, 15 rats each; Group I received the respective solvents in a volume of 0.2 ml/200 g animal body weight and served as a control group. Group II received 0.25% CMC as vehicle and 3-NP (10 mg/kg/day; i.p) [36]. Group III was treated with PIO (40 mg/kg/day; p.o.) [19] and 3-NP. Group IV was treated with PIO and 3-NP as above, as well as the PPARγ antagonist, GW9662 (1 mg/kg/day; i.p.) [35]. Group V was treated with TEL (10 mg/kg/day; p.o**.**) [35, 37] and 3-NP. Group VI was treated with TEL and 3-NP as above, as well as GW9662. 3-NP was dissolved in saline, whereas PIO and TEL were suspended in 0.25% CMC solution. GW9662 was dissolved in 50% DMSO, freshly diluted with saline, and administered concomitantly with PIO or TEL. Vehicle, PIO, or TEL were administered 1 h before the neurotoxin injections, and all treatments were administered for 14 days (Fig. 1).

After 14 days, behavioral anomalies were assessed using the open field, rotarod, and novel object recognition tests. Then, animals were killed by cervical dislocation under anesthesia (Fig. 1). Subsequently, brains were rapidly exposed and washed with ice-cold saline. Accordingly, animals in each group were further divided into 3 sets: first set (n=3): brains were promptly fixed in 10% (v/v) buffered formalin for 72 h for the histopathological and immunohistochemistry techniques. In the other sets (n=6, each), striata were excised from each rat on an ice-cold glass plate and then stored at −80 °C to perform biochemical analyses. In the second set, the striata were homogenized in ice-cold phosphate-buffered saline (PBS) for enzyme-linked immunosorbent assay (ELISA) analyses. In the third set, one striata from each rat were used to measure parameters by Western blot, and the other striata were used for qRT-PCR. Animals were assigned into groups by a technical assistant in a blinded manner. As well, assessment of all measurements of the study, sample coding, and decoding was done by a researcher blinded to the sample identity.

## Measurement of Body Weight

Animal body weights (BW) were recorded on the first (1^st^ day) and last day of the study (15^th^ day). The following formula was used to calculate the percent change of body weight.

$$\mathrm{\%\;Change\;in\;body\;weight}=\frac{\mathrm{BW\;on }{1}^{\mathrm{st}}\mathrm{\;day}-\mathrm{BW ON }{15}^{\mathrm{th}}\mathrm{ day}}{\mathrm{BW\;on\;}1\mathrm{st\;day}}\times 100$$

### Behavioral Evaluation

#### Open Field Test

Open field test was performed to assess the rats’ spontaneous exploratory activity. The open field apparatus was made up of wood and had dimensions of 100 x 100 x 40 cm, divided into 16 rectangular squares, and videotaped for the 3-min recording session. The experimental room was illuminated by a 40-watt white bulb positioned 150 cm above the test apparatus. Each rat was gently placed in the center of the floor, and behavioral activity (number of squares crossed, locomotion, rearing, grooming, and latency time) was recorded. Each crossing was considered only when all four paws moved to another square [36].

#### Rotarod Activity

Rotarod test was used to monitor fore- and hind-limb motor coordination and balance. In brief, the rotarod apparatus comprises a rod of 120 cm in length and 7 cm in diameter and operates at a constant speed of 25 rpm (IMCORP, Ambala, India). Before starting the experiment, animals were trained for three consecutive days, with three trials/day. Animals that failed to be well balanced or showed no attempt to run were excluded. During the training sessions, animals were returned to the rotarod each time they fell off the rod. The mean of three latencies to fall was calculated for each rat to yield a final value, and the cutoff time was 300 s [38].

#### Novel Object Recognition Test

The novel object recognition (NOR) test is an efficient tool for testing memory and learning in rats [39]. It relies on the animal's natural preference for novelty in its environment. The task procedure was accomplished over three successive days. On the first day (habituation), rats were placed in the center of a wooden box (40 cm × 40 cm × 40 cm) individually for 10 min to explore the environment with no objects placed. On the second day (training), rats were placed individually in the box for 10 min, but the animals were allowed to explore two similar objects (rectangles 7 × 5 × 10 cm, positioned 7 cm away from the walls of the apparatus). On the third day (test), one of the explored objects was substituted by a new object (cylinder 7 × 5 cm), and each animal was allowed to explore the objects in the box for 3 min. After testing each rat, the objects and box walls were thoroughly cleaned with 70% ethanol to remove any residual olfactory cues. The time consumed by each rat exploring (when the rat sniffed or looked at the objects from no more than 2 cm away) the objects at the new (novel) and/or the old (familiar) locations were recorded, and the following parameters were computed:

1. Discrimination index (DI): Difference between the time exploring the novel and the familiar objects divided by the total time spent exploring both objects.
2. Recognition index (RI): Time consumed by the animal exploring the novel object as a percentage of the total exploration time.

## Biochemical Measurements

### Enzyme-Linked Immunosorbent Assay (ELISA)

Striatal levels of tumor necrosis factor-alpha (TNF-α) (Cat. no. RTA00), interleukin 1β (IL-1β) (Cat. no. RLB00), interleukin-6 (IL-6) (Cat. no. R6000B) (R&D Systems Inc., Minneapolis, USA), Adenosine triphosphate (ATP) (Cat. no. MBS723034), and succinate dehydrogenase (SDH) (Cat. no. MBS3807968) (MyBioSource, San Diego, CA, USA) were measured using Rat ELISA kits according to the manufacturer’s instructions.

### Western Blot Analysis

Protein solutions were isolated from striatal tissues. Then, equal amounts of proteins were loaded onto a sodium dodecyl sulfate–polyacrylamide (SDS-PAGE) gel electrophoresis, to be separated according to their molecular weight. Subsequently, the samples were electrotransferred onto vinylidene difluoride membranes (Pierce, Rockford, IL, USA), using an electroblotting apparatus (Bio-Rad, Hercules, CA, USA). These membranes were blocked with 10% skim milk in Tris-buffered saline for 1 h at room temperature to prevent nonspecific binding. Then, membranes were incubated overnight at 4˚C with one of the following primary antibodies: pS473 Akt-1, pS9 GSK-3β, cleaved caspase-3, pS536 p65 NF-κB or β-actin (Cell Signaling Technology, Boston, MA). The immunoreactive bands were demonstrated by incubation with horseradish peroxidase (HRP)-labeled secondary antibody (Santa Cruz Biotechnology) at room temperature for 1 h. Lastly, peroxidase activity was visualized with an enhanced chemiluminescence reagent kit (Amersham Biosciences, Piscataway, NJ, USA). Results were expressed as arbitrary units after normalization with ß-actin protein expression.

### Quantitative Real-Time Polymerase Chain Reaction (qRT-PCR)

Total RNA was extracted from striatal tissue samples using Qiagen tissue extraction kit (Qiagen, USA) following the manufacturer's instructions. The purity and concentration of RNA were determined spectrophotometrically at 260/280 nm. Subsequently, RNA was reverse-transcribed into first-strand complementary DNA (cDNA) using a high-capacity cDNA synthesis kit (Thermo Fisher Scientific Co., USA) according to the manufacturer’s procedure. The amplification process was conducted using SYBR Green JumpStart Taq ReadyMix (Sigma-Aldrich, St. Louis, MO, USA) in accordance with the manufacturer’s protocol. The gene-specific primers used for PCR analysis are listed in Table 1. The relative amount of the target mRNA was obtained using the 2 − ΔΔCT method and normalized to ß-actin [40].

**Table 1:** The sequence of primers used for real-time PCR analysis

Gene	Primer sequence
PPARγ	F: 5′-TGA TAT CGA CCA GCT GAACC-3′ R: 5′-GTC CTC CAG CTG TTC GCCA-3′
PGC-1α	F: 5′-GCA CCA GAA AAC AGC TCC AA-3′ R: 5′-TTG CCA TCC CGT AGT TCA CT-3′
TFAM	F: 5′-ATC AAG ACT GTG CGT GCA TC-3′ R: 5′-AGA ACT TCA CAA ACC CGC AC-3′
Bcl-2	F: 5′-TGT GGA TGA CTG ACT ACC TGA ACC-3′ R: 5′-CAG CCA GGA GAA ATC AAA CAG AGG-3′
IL-10	F: 5′-CAC CAC CCT CCT TGT TCA AC-3′ R: 5′-CAA TCC ACA ACT CGC TCC AA-3′
β-actin	F: 5′- TCT TCC AGC CTT CCT TCC TG-3′ R: 5′-CAA TGC CTG GGT ACA TGG TG-3′

### Hematoxylin and Eosin (H&E) Staining

A histological assessment was performed on the brain of three rats randomly selected from each group. Rat brain tissue samples were dissected and placed in 10% buffered formalin for 72 h. The specimens were passed through serial grades of ethanol, cleared in xylene, and embedded in paraffin at 56 °C in a hot air oven for 24 h. Paraffin bees wax tissue blocks were prepared for sectioning at 5 μm thickness by rotatory microtome for demonstration of striatal regions in different samples. The obtained tissue sections were collected on glass slides, deparaffinized, stained by H&E stain, coverslipped, and then examined through the light electric microscope.

### Cresyl Violet (Nissl) Staining

Nissl staining is a histological method that identifies neurons with Nissl’s substance and indicates healthy tissues. Paraffin sections of 5 μm thickness were mounted on slides and then stained with Cresyl violet dye (1% w/v in water) for 5 min and air-dried at room temperature for 1 h. Then, the sections were cleared with xylene, coverslipped, and examined microscopically. The average number of intact neurons was calculated across six randomly selected fields for each animal using the Leica Qwin software. Only neurons with a visible nucleus and with an apparent entire outline were considered normal and were counted.

### Immunostaining for Striatal Microglial Marker

For immunohistochemical analysis, brain pieces were processed into paraffin blocks; thereafter, 5-μm sections were fixed into positively charged glass slides. Endogenous peroxidase activity was quenched by first incubating the specimens in 3% hydrogen peroxide for 15 min. The specimens were then incubated with anti-ionized calcium-binding adapter molecule 1 (Iba-1) antibody (diluted 1:1000; cat. no. ab184787, Abcam, Cambridge, MA, USA), followed by sequential incubations with biotinylated link antibody and peroxidase-labeled streptavidin (Dako, Carpinteria, CA, USA). Labeling was then revealed by incubation with diaminobenzidine for 10 min. Slides were counterstained with hematoxylin, dehydrated, coverslipped, and examined through the light electric microscope [Olympus CX21, Tokyo, Japan]. Six random striatal non-overlapping fields were scanned and analyzed for determining the mean number of Iba-1^++^ microglial cells in each immunostained section. Morphological examination, photographs, as well as quantitative analysis were determined microscopically (magnification × 40) using the Leica Qwin 500 Image Analyzer (Leica Microsystems, Wetzlar, Germany).

### Determination of Protein Content

Protein content was assessed according to the method of Lowry et al. [41].

#### Statistical Analysis

Initially, all results were tested for normality using the Kolmogorov–Smirnov test. All values seemed to be normally distributed and were presented as mean ± S.D. Data were analyzed by one-way analysis of variance (ANOVA) followed by Tukey's multiple comparison tests using Prism8 (Graph Pad Software, San Diego California, USA). A probability level of < 0.05 was accepted as statistically significant.

## Results

### Effect of PIO, TEL, With and Without GW9662 on (3-NP)-Induced Motor Deterioration

**Fig. 2:**
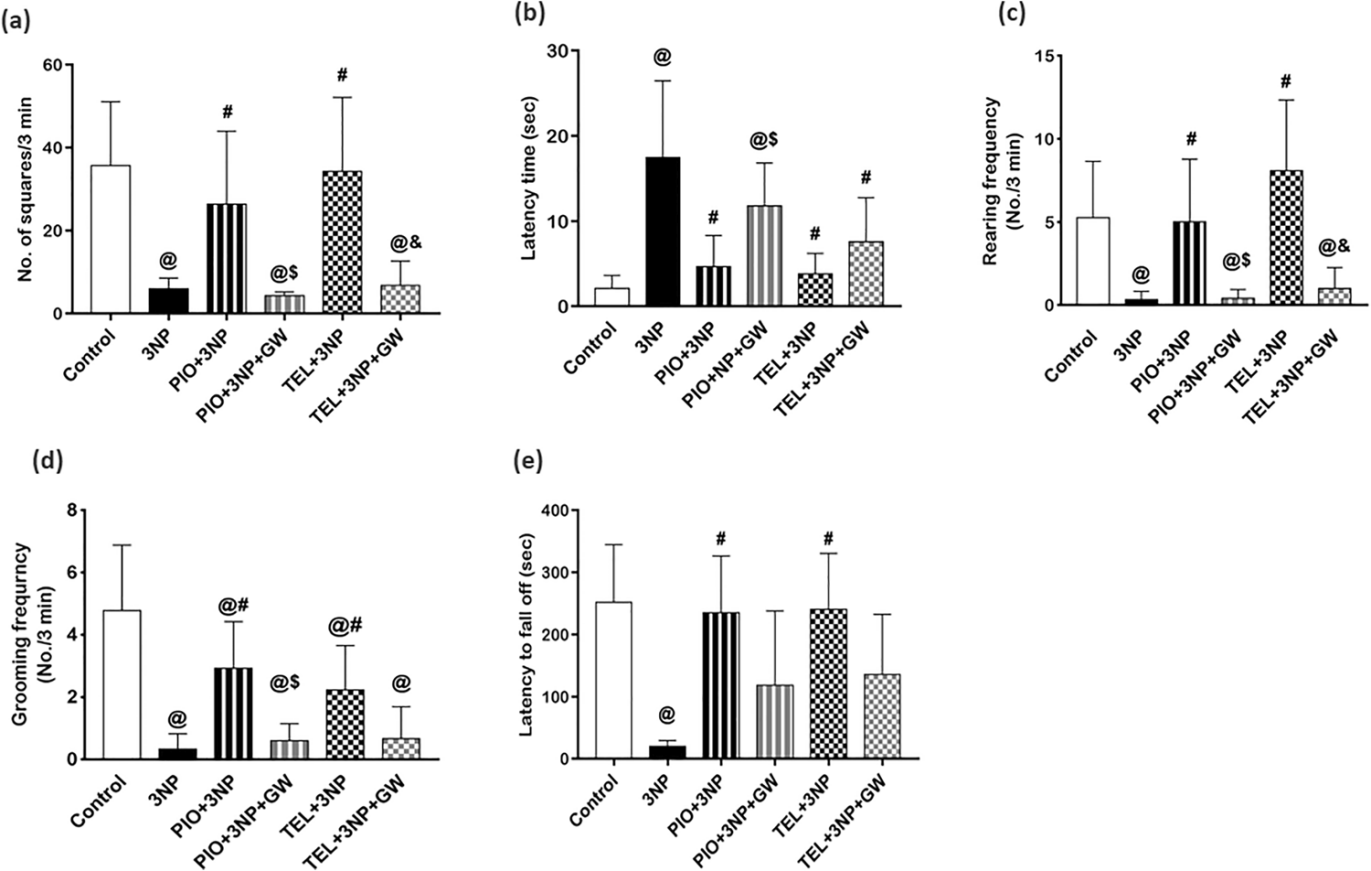
Effects of PIO and TEL with and without PPARγ blocker on (**a**) number of squares, (**b**) latency time, (**c**) rearing frequency, (**d**) grooming frequency, and (**e**) fall-off time in 3-NP-treated rats. Data are presented as means ± SD (*n* = 15) and analyzed by one-way ANOVA followed by the Tukey’s post hoc test. @ *vs* control, # *vs* 3-NP group, $ *vs* PIO-pre-treated group, & *vs* TEL-pre-treated group. (PIO: pioglitazone; TEL: telmisartan; 3-NP: 3-nitropropionic acid; GW: GW9662)

Continuous 3-NP administration for 14 days showed behavioral and motor abnormalities in the experimental rats as manifested by open field and rotarod tests. In the open field test, a significant decrease in ambulation (by 83.2%), rearing (by 93.7%), and grooming (by 93%) frequencies, alongside a marked increase in latency time (8.3-fold), were observed among the 3-NP group as compared to the normal control group (Fig. 2a,b,c,d). The retention time on a rotating rod was also found to be significantly reduced (by 91.8%) in 3-NP-treated rats compared to the control group (P < 0.05) (Fig. 2e).

PIO treatment showed an increase in ambulation, rearing, and grooming frequencies to reach approximately 4.4-, 15-, and 8.8-fold, respectively, as compared to the 3-NP control group (*P* < 0.05). Moreover, there was an approximate normalization of the falling time values in the rotating rod, as well as an obvious decline in latency time by 73.3% after treatment with PIO compared to the 3-NP control rats (P < 0.05).

Additionally, treatment with TEL significantly succeeded to increase rearing and grooming frequencies to reach approximately 24.2- and 6.7-fold, respectively, versus the 3-NP control group (*P* < 0.05), and approximately normalized the ambulation frequency and fall-off time. As well, there was a significant decrease in latency time by 77.8% in TEL-pretreated rats compared to 3-NP treated rats (*P* < 0.05).

The addition of the PPARγ blocker, GW9662, to PIO-treated rats decreased ambulation (by 83.9%), rearing (by 92%), and grooming (by 79.5%) frequencies, and halved the fall-off time, while the latency time value was increased by 2.5-fold as compared to PIO-treated rats (*P* < 0.05). The effect on fall-off time is non-significant, when compared to PIO-treated group (*P* < 0.05). Also, administration of GW9662 to the TEL group partially abolished such improvement in motor performance and decreased ambulation (by 80%), rearing (by 87.6%), grooming (by 70.1%) frequencies, and fall-off time (by 43.6%). Further, the latency time value was nearly doubled. The effect on latency time, fall-off time, and grooming is considered non-significant, when compared to the TEL-treated group (*P* < 0.05).

### Effect of PIO, TEL, With and Without GW9662 on (3-NP)-Induced Cognitive Decline and Weight Loss

**Fig. 3:**
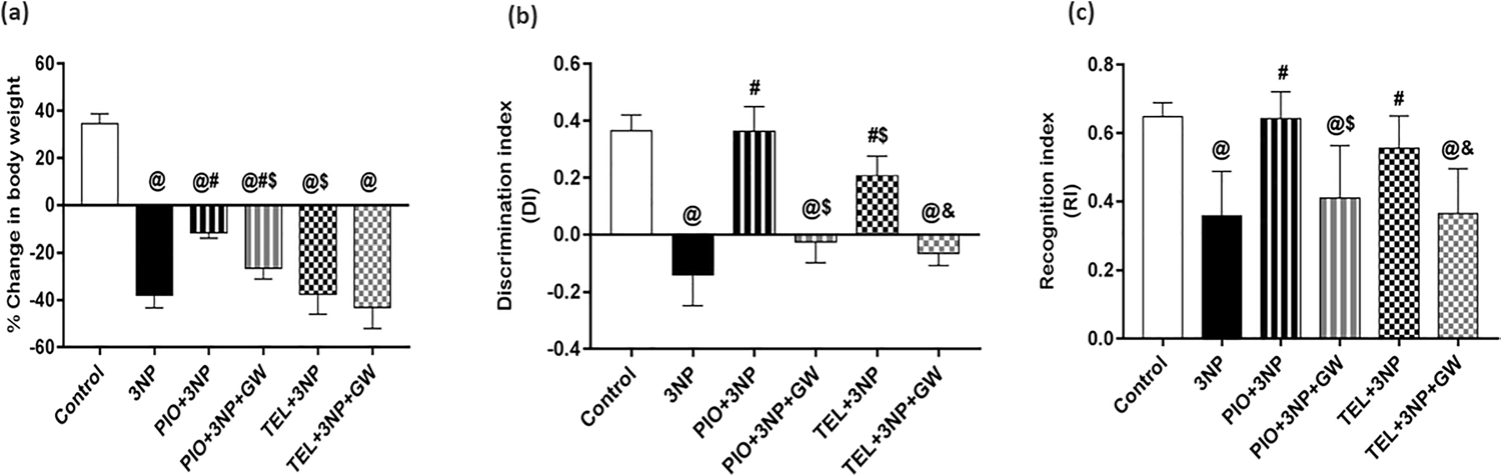
Effects of PIO and TEL with and without PPARγ blocker on (**a**) body weights, (**b**) discrimination index, and (**c**) recognition index in 3-NP-treated rats. Data are presented as means ± SD (*n* = 15) and analyzed by one-way ANOVA followed by the Tukey’s post hoc test. @ *vs* control, # *vs* 3-NP group, $ *vs* PIO-pre-treated group, & *vs* TEL-pre-treated group. (PIO: pioglitazone; TEL: telmisartan; 3-NP: 3-nitropropionic acid; GW: GW9662)

As shown in Fig. 3a, there was a significant drop (by 219%) in the body weight of 3-NP treated rats when compared with the control rats (P < 0.05). PIO prevented this fall in body weight and achieved a significant change (3.2-fold) with respect to 3-NP treated group (P < 0.05). The addition of GW9662 significantly reversed this gain in body weight. On the contrary, TEL treatment did not cause any further change in body weight, compared to the 3-NP group (P < 0.05). 3-NP administration detracted discrimination (DI) and recognition (RI) indices in the novel object recognition test by 138.4% and 44.8%, respectively, as compared to the normal control group. PIO approximately normalized these values, whereas TEL attained a 2.5- and 1.2-fold increase in the DI and RI values, respectively, as compared to the 3-NP group and showed a significant difference (for DI) when compared with PIO-treated animals (P < 0.05). Interestingly, co-treatment with GW9662 partially inhibited this observed improvement of cognitive decline (Fig. 3b,c).

### Effect of PIO, TEL, With and Without GW9662 on (3-NP)-Induced Decline of PPARγ, PGC-1α, TFAM, SDH (Complex-II), and ATP

**Fig. 4:**
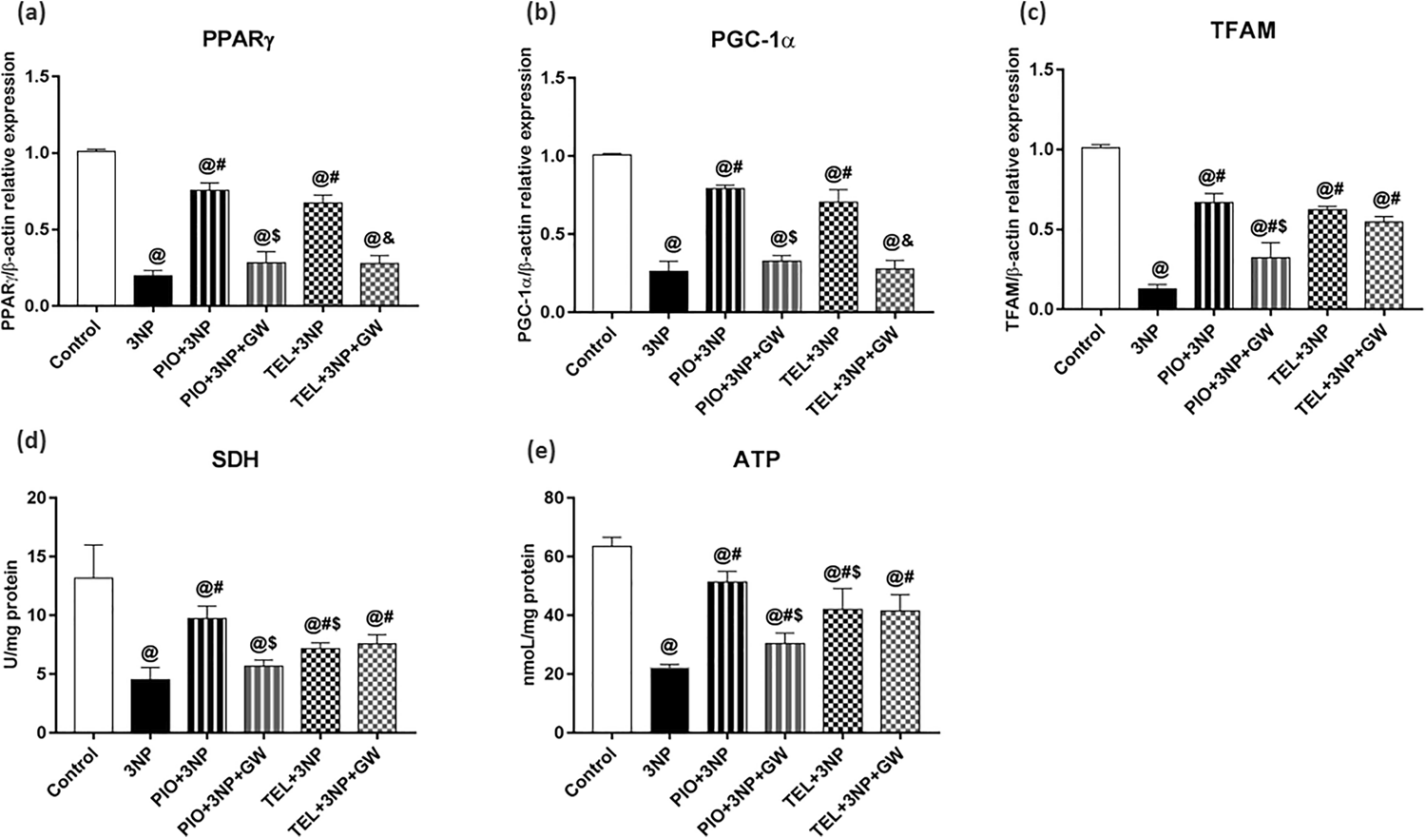
Effects of PIO and TEL with and without PPARγ blocker on striatal gene expressions of (**a**) PPARγ, (**b**) PGC-1α, and (**c**) TFAM, as well as striatal levels of (**d**) SDH, and (**e**) ATP in 3-NP-treated rats. Data are presented as means ± SD (*n* = 6) and analyzed by one-way ANOVA followed by the Tukey’s post hoc test. @ *vs* control, # *vs* 3-NP group, $ *vs* PIO-pre-treated group, & *vs* TEL-pre-treated group. (PIO: pioglitazone; TEL: telmisartan; 3-NP: 3-nitropropionic acid; GW: GW9662)

An important metabolic feature that characterizes the HD model is the aberrant mitochondrial function. 3-NP showed a significant impairment of the mitochondrial respiratory chain by reducing the transcript level of striatal PPARγ and its downstream target genes PGC-1α and TFAM to 20, 26, and 13% of control values, respectively (P < 0.05) (Fig. 4a,b,c). As well, the activity of striatal mitochondrial SDH and level of ATP declined to reach 34 and 35% of the control rat's values, respectively (P < 0.05) (Fig. 4d,e).

The mitochondrial energy production was greatly improved in PIO- and TEL-treated groups as depicted by enhanced PPARγ (3.8-fold [PIO] and 3.4-fold [TEL]), PGC-1α (threefold [PIO] and 2.7-fold [TEL]), TFAM (5.2-fold [PIO] and 4.9-fold [TEL]) expressions as well as activity of SDH (2.2-fold [PIO] and 1.6-fold [TEL]), and ATP level (2.3-fold [PIO] and 1.9-fold [TEL]) significantly, concerning rats that received 3-NP (P < 0.05). Notably, PIO was greater than TEL in enhancing mitochondrial ATP and SDH, and this difference appears to be statistically significant (P < 0.05).

The recovery produced by PIO was dampened in the GW9662-cotreated animals and decreased by 62.4, 58.6, 51.6, 51.6, and 59.2% of the PIO-treated group, regarding PPARγ, PGC-1α, TFAM, SDH, and ATP, respectively.

Improvement by TEL was abolished by the addition of GW9662 regarding PPARγ and PGC-1α to reach 41.5 and 39.3%, respectively, as compared to the 3-NP + TEL group. However, mitochondrial TFAM, SDH, and ATP were not different from the 3-NP + TEL group (P < 0.05), indicating that such enhancement of mitochondrial parameters was not attributed to the PPARγ activity of TEL.

### Effect of PIO, TEL, With and Without GW9662 on (3-NP)-Precipitated Inflammatory Response (NF-κB p65 (pS536), TNF-α, IL-1β, IL-6, IL-10)

It is commonly known that activating the NF-κB subunit plays a crucial role in regulating the expression of proinflammatory cytokines.

3-NP significantly boosted the expressions of NF-κB by 6.4-fold, as well as levels of TNF-α, IL-1β, and IL-6 by 3.3-, 4.3-, and 3.8-fold, respectively, above their normal values. In addition, striatal IL-10 expression was significantly declined after 3-NP injections to near 25% of their control group values (P < 0.05).

**Fig. 5:**
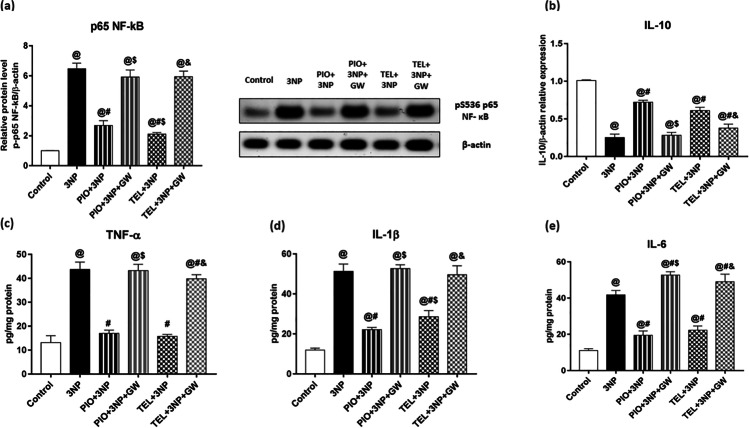
Effects of PIO and TEL with and without PPARγ blocker on striatal protein/gene expressions of (**a**) p65 NF- κB and (**b**) IL-10, as well as striatal levels of (**c**) TNF-α, (**d**) IL-1β, and (**e**) IL-6 in 3-NP-treated rats. Data are presented as means ± SD (*n* = 6) and analyzed by one-way ANOVA followed by the Tukey’s post hoc test. @ *vs* control, # *vs* 3-NP group, $ *vs* PIO-pre-treated group, & *vs* TEL-pre-treated group. (PIO: pioglitazone; TEL: telmisartan; 3-NP: 3-nitropropionic acid; GW: GW9662)

Significantly, PIO and TEL pre-treatment reduced the expression of NF-κB (by 58.6% [PIO] and by 67.4% [TEL]), and its downstream inflammatory markers, TNF-α (by 61.2% [PIO] and by 64% [TEL]), IL-1β (by 56.9% [PIO] and by 44.3% [TEL]) and IL-6 (by 53.2% [PIO] and by 46.6% [TEL]) in comparison with 3-NP rats (P < 0.05). Besides, both compounds significantly augmented the anti-inflammatory IL-10 expression to attain a 2.9 [PIO] and 2.4 [TEL]-fold increase as compared to 3-NP rats (P < 0.05). Further improvement by PIO and TEL was abolished by the addition of GW9662 (Fig. 5).

### Effect of PIO, TEL, With and Without GW9662 on (3-NP)-Induced Striatal Neuronal Apoptosis (pAkt (Ser 473), pGSK-3β (Ser 9), Bcl-2, Cleaved Caspase 3)

**Fig. 6:**
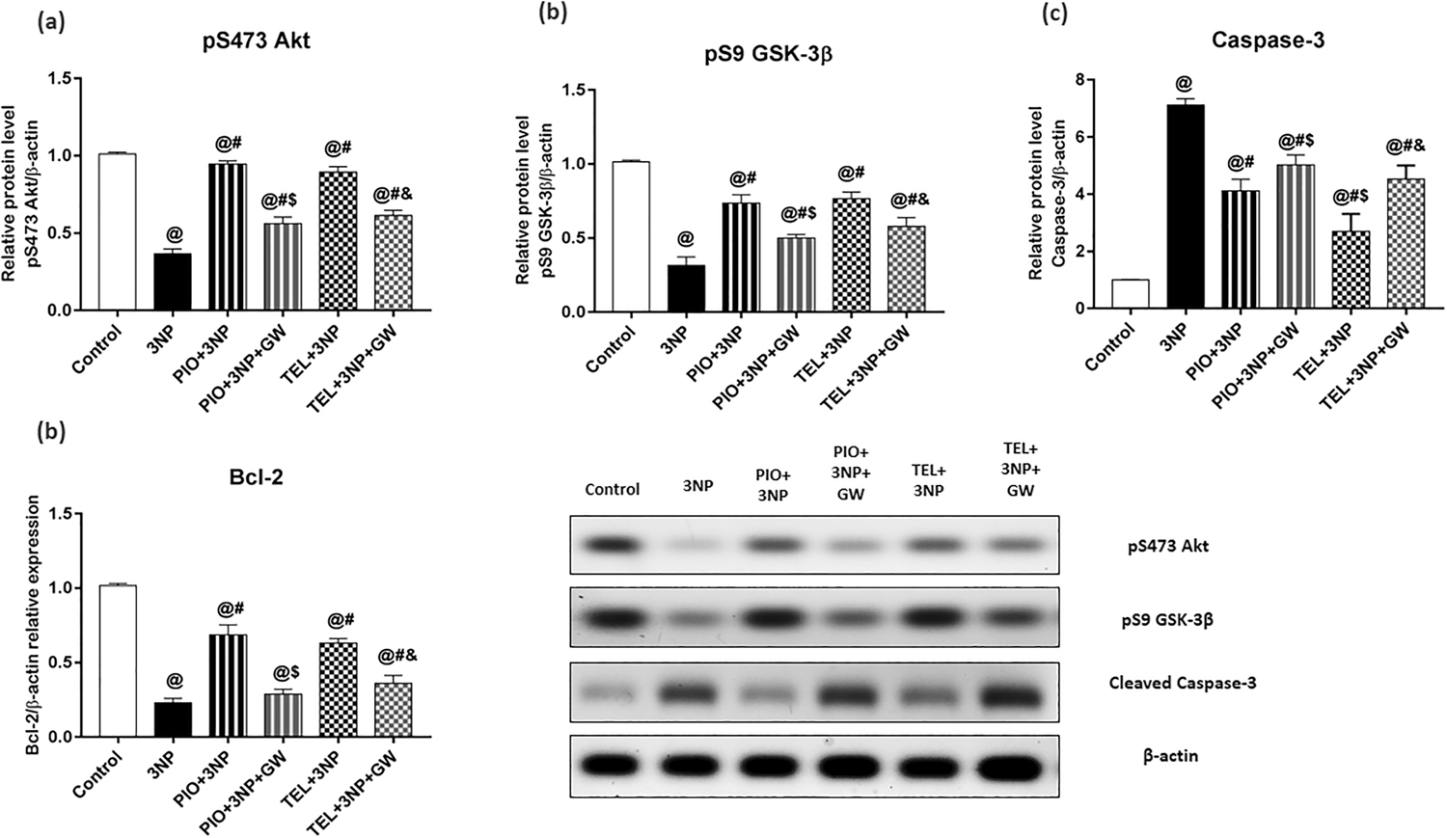
Effects of PIO and TEL with and without PPARγ blocker on striatal protein/gene expression of (**a**) pS473 Akt, (**b**) pS9 GSK-3β, (**c**) Cleaved caspase-3, and (**d**) Bcl-2 in 3-NP-treated rats. Data are presented as means ± SD (*n* = 6) and analyzed by one-way ANOVA followed by the Tukey’s post hoc test. @ *vs* control, # *vs* 3-NP group, $ *vs* PIO-pre-treated group, & *vs* TEL-pre-treated group. (PIO: pioglitazone; TEL: telmisartan; 3-NP: 3-nitropropionic acid; GW: GW9662)

3-NP injection mitigated expressions of striatal pAkt, pGSK-3β, and Bcl-2 by 63.9, 68.9, and 77.3%, respectively, while increased the protein expression of caspase-3 by 7.1-fold if compared to the control group values (P < 0.05). Interestingly, PIO amended the alterations of pAkt, pGSK-3β, and Bcl-2 and achieved a 2.6-, 2.3-, and 3-fold increase, respectively, and abolished such elevation of caspase-3 by 36%, respectively, of the 3-NP animal group (Fig. 6).

These findings with PIO, however, were inundated in GW9662-cotreated rats and decreased by 40.7, 32.1, 58% regarding pAkt, pGSK-3β, and Bcl-2, respectively. Besides, the inhibitory effect of PIO on caspase-3 protein expression was diminished and increased by about 21.9%, as compared to PIO-treated animals.

Similarly, TEL elevated pAkt, pGSK-3β, and Bcl-2 by 2.4-, 2.4-, and 2.7-fold, respectively, and lowered the protein expression of caspase-3 to reach 37.9% of the 3-NP group.

As described in Fig. 6, GW9662 significantly erased the protective effect of TEL and decreased the expressions of pAkt by 31.4%, pGSK-3β by 24.2, and Bcl-2 by 42.5%, while increased caspase-3 expression by approximately 68%, as opposed to rats not co-treated with GW9662.

### Effect of PIO, TEL, With and Without GW9662 on (3-NP)-Induced Histological Changes

**Fig. 7:**
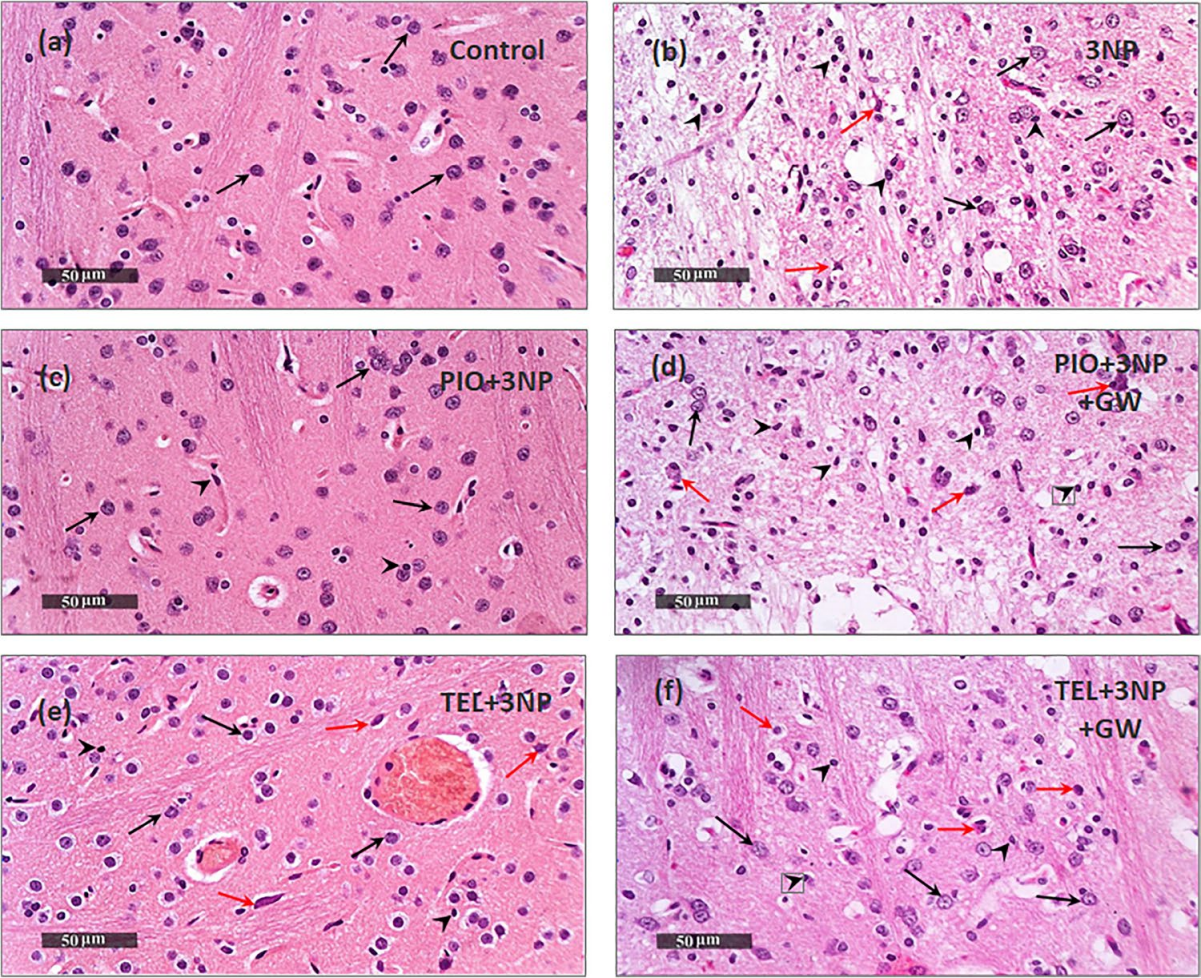
Effects of PIO and TEL with and without PPARγ blocker on histopathological alterations in 3-NP-treated rats. Representative H&E-stained striatal sections: (**a**) control, (**b**) 3NP group, (**c**) PIO-pre-treated group, (**d**) PIO&GW-pre-treated group, (**e**) TEL-pre-treated group, and (**f**) TEL&GW-pre-treated group. Black arrows indicate intact neurons, red arrows indicate necrotic neurons, while black heads indicate microglial infiltrates

**Fig. 8:**
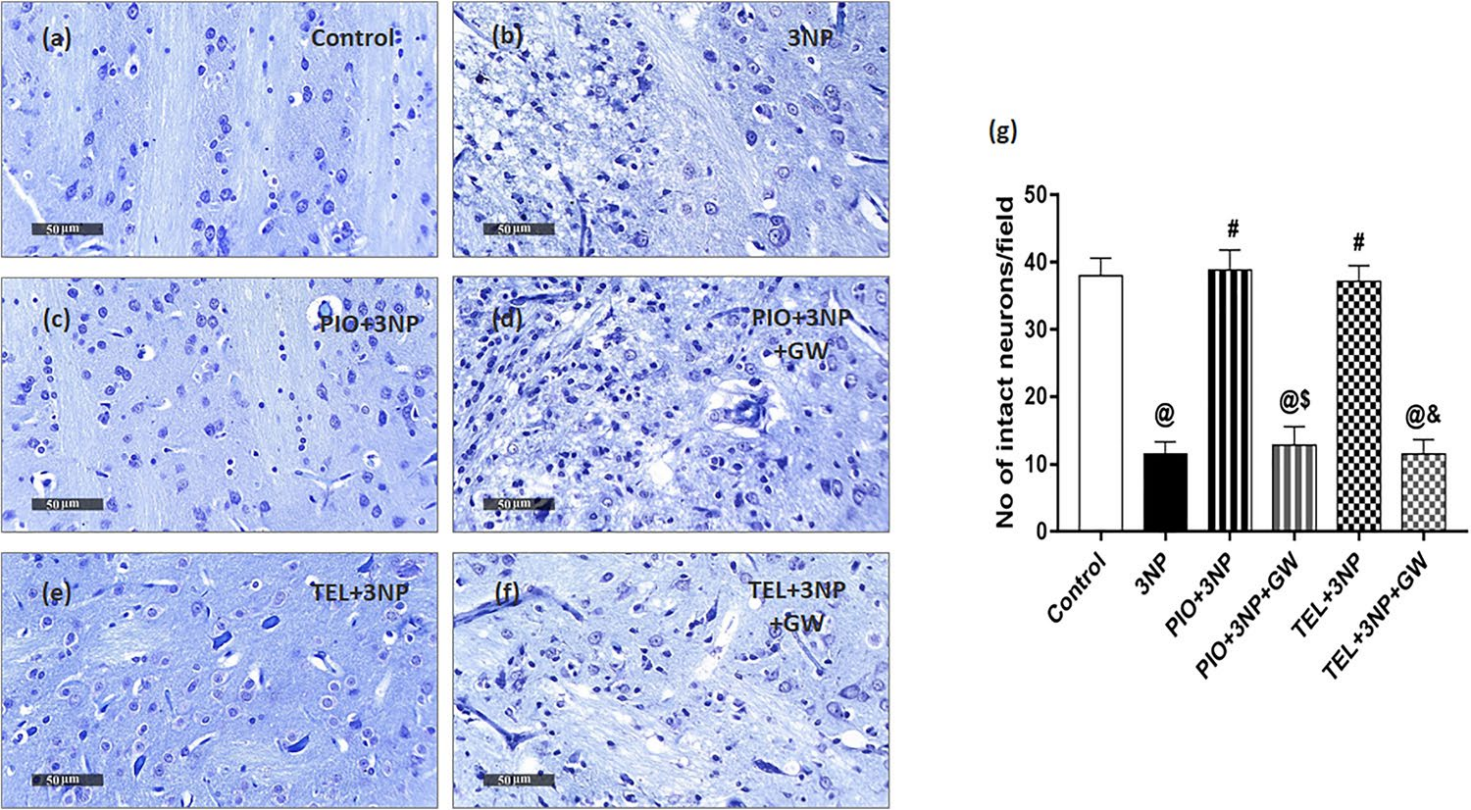
Effects of PIO and TEL with and without PPARγ blocker on neuronal survival in 3-NP rats. Representative cresyl violet-stained striatal sections: (**a**) control, (**b**) 3NP group, (**c**) PIO-pre-treated group, (**d**) PIO&GW-pre-treated group, (**e**) TEL pre-treated group, and (**f**) TEL&GW-pre-treated group. (**g**) Quantification of intact neurons in striatal sections of the experimental groups. Data are presented as means ± SD (*n* = 3) and analyzed by one-way ANOVA followed by the Tukey’s post hoc test. @ *vs* control, # *vs* 3-NP group, $ *vs* PIO-pre-treated group, & *vs* TEL-pre-treated group. (PIO: pioglitazone; TEL: telmisartan; 3-NP: 3-nitropropionic acid; GW: GW9662)

The most striking hallmark of HD is the preferential loss of striatal neurons. Neuroprotection afforded by PIO and TEL was assessed through histopathologic examination of H&E-stained sections, as well as, quantifying the number of striatal viable neurons using Nissl stain (Figs. 7 and 8). Control samples showed robust morphological features of the striatum with apparent intact well-organized neurons presenting intact subcellular and nuclear details (black arrow). Intact intercellular brain matrix with few records of reactive glial cell infiltrates was observed. On the contrary, photomicrographs from 3-NP rats demonstrated a focal area at the anterior lateral border of striatum regions with noticeable neuronal loss and many degenerated neurons, portended by a 69.7% decline in viable neurons, along with the persistence of a few degenerated and necrotic small neurons (red arrow). Moderate edema and vacuolization of the brain matrix accompanied by many reactive microglial cells infiltrates (arrowhead) were shown in the lesion core. Intact neurons were demonstrated at lesion borders (black arrows). In comparison with the 3-NP group (P < 0.05), rats treated by PIO showed preserved striatal architecture with many apparent intact homogenously distributed neurons (black arrows) and minimal degenerated shrunken cells, represented by a 3.4-fold increase in intact neurons. An intact intercellular brain matrix with significantly few records of microglial infiltrates was observed (arrowhead). Similarly, the TEL group showed mild few scattered records of neuronal degenerative changes (red arrow) with mild perineuronal edema. However, most of the neurons demonstrated apparent intact subcellular details (black arrow) with a significant reduction of reactive microglial cells infiltrates and achieved a 3.2-fold increase in intact neurons as compared to the 3-NP group (P < 0.05). Pre-administration of PPARγ inhibitor showed less improvement than that seen with PIO- or TEL-treated rats with significant neuronal loss and reactive glial cells infiltrates.

### Effect of PIO, TEL, With and Without GW9662 on (3-NP)-Induced Microglial Activation

**Fig. 9:**
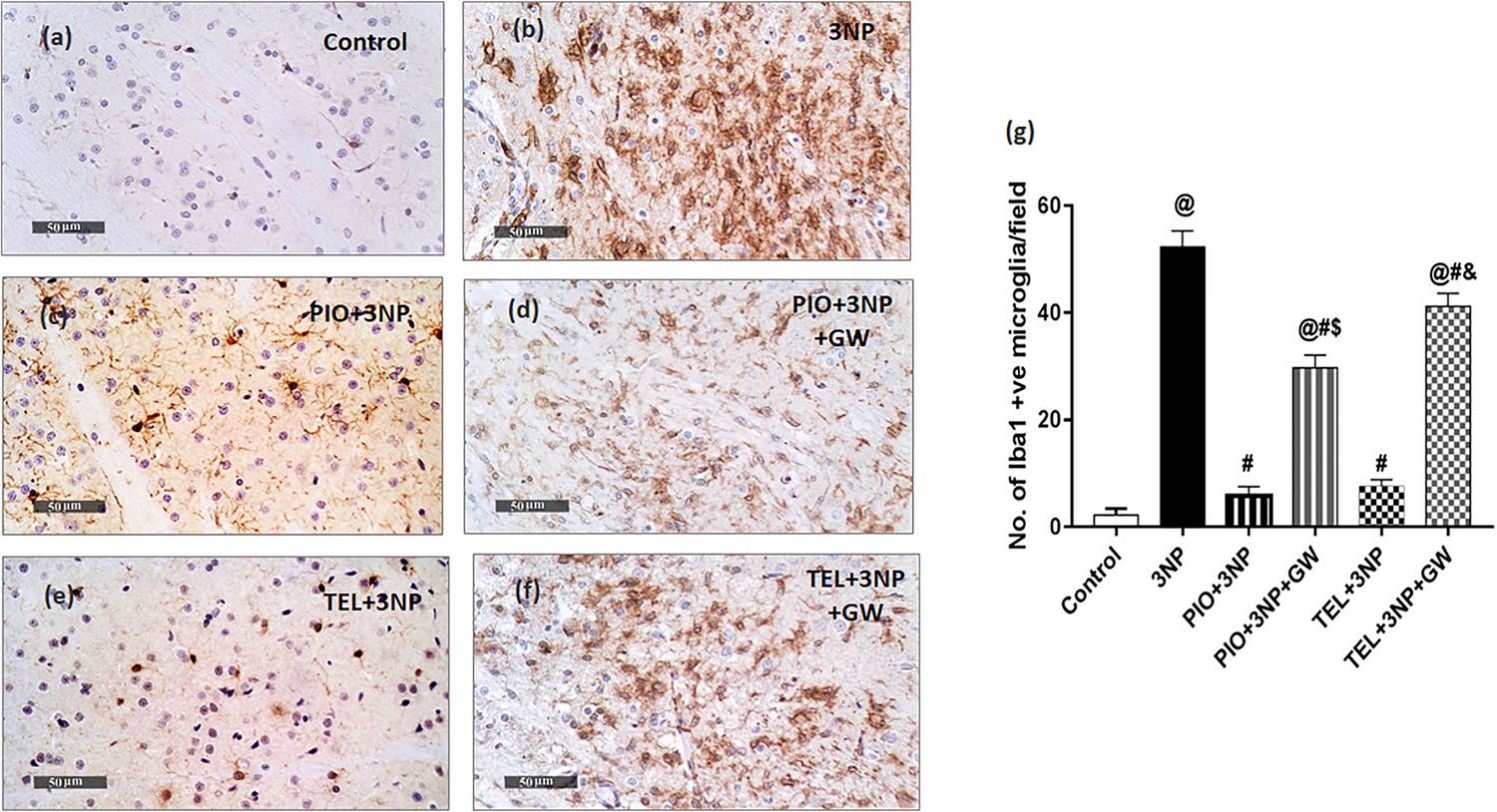
Effects of PIO and TEL with and without PPARγ blocker on immunoreactivity of Iba-1^+^ in the striatum of 3-NP-treated rats. (**a**) Control, (**b**) 3-NP group, (**c**) PIO-pre-treated group, (**d**) PIO&GW-pre-treated group, (**e**) TEL-pre-treated group, and (**f**) TEL&GW-pre-treated group. (**g**) Quantification of Iba-1-positive microglial cells in striatal sections of the experimental groups. Data are presented as means ± SD (*n* = 3) and analyzed by one-way ANOVA followed by the Tukey’s post hoc test. @ *vs* control, # *vs* 3-NP group, $ *vs* PIO-pre-treated group, & *vs* TEL-pre-treated group. (PIO: pioglitazone; TEL: telmisartan; 3-NP: 3-nitropropionic acid; GW: GW9662)

A central response to inflammatory brain insults is the activation and proliferation of microglia. Ionized calcium-binding adaptor molecule-1 (Iba-1) is a calcium-binding protein that occurs only on the microglial surface. Immunohistochemical examination demonstrated that the average number of striatal Iba-1^+^ microglia was greatly higher (26-fold) among rats that received 3-NP as compared to the control rats (p < 0.05) (Fig. 9). Conversely, the mean number of Iba-1^+^ cells decreased significantly in the 3-NP + PIO group (by 88.9%) and 3-NP + TEL group (by 85.4%) versus 3-NP lesioned rats (p < 0.05). Further improvement by PIO and TEL was revoked by the addition of GW9662 (Fig. 9).

## Discussion

The present work reveals the neuroprotective action of TEL in the 3-NP rat model of HD. TEL is known to act, not only as an AT1R blocker but also as a partial activator of PPARγ [25], whose activation is correlated with the enhancement of mitochondrial function as well as modulation of glial and neuroinflammatory response [42]. To uncover the role of PPARγ activation in the TEL neuroprotection, we inspected the effects of the full PPARγ agonist, PIO, and the PPARγ antagonist, GW9662.

The principal observations in this study are: (**1)** 3-NP-treated group exhibited low mRNA expression of PPARγ and its downstream PGC-1α, the key regulator of the cell energy machinery. (**2)** The resulting energy deficiency, as well as motor and cognitive impairment, were effectively ameliorated by TEL. **(3)** TEL abrogates 3-NP-induced striatal microglial activation and the subsequent release of pro-inflammatory molecules. (**4)** The boost in IL-10 expression by TEL may contribute to additional anti-inflammatory action. **(5)** TEL neuroprotection is associated with the prevention of 3-NP-induced inhibition of Akt/GSK3β phosphorylation, suppressing neuronal apoptosis and restoring striatal neuron integrity. **(6)** Most of the beneficial effects of TEL are PPARγ dependent and comparable to that of PIO.

The most affected brain region in HD is the striatum, an information-processing center of the basal ganglia, which receives inputs from the motor and cognitive cortical areas to execute and organize a behavioral output [43]. As depicted in our study, systemic 3-NP (10 (mg/kg)/day, for 2 weeks) in rats significantly induced HD-like symptoms, manifested by impaired locomotion, motor coordination, and cognitive function. Our observations stay in line with several studies representing similar behavioral and cognitive perturbations following 3-NP intoxication [36, 38, 44–48]. These observations point to the neurotoxic capability of this model to imitate behavioral signs observed especially in the later stage of HD, where dystonia and poor motor coordination predominate. Receptors of PPARγ were found to be highly expressed in some brain regions including the striatum [49]. In this respect, PIO as a PPARγ agonist limited motor impairment in a rat model of HD induced by the neurotoxin, quinolinic acid, and this effect was reversed by a PPARγ antagonist [19]. Besides, it was recorded to improve motor and coordination abilities in animal models of PD [50, 51]. Similarly, TEL enhanced the neurobehavioral performance in rat models of neurovascular impairment [52], depression [53], PD [54], and cerebral ischemia [55]. These previous publications coincide with our observation where PIO and TEL reversed the effects of 3-NP on locomotion and rotarod performance. Additionally, we observed a significant improvement in cognitive function after PIO treatment as shown in the NOR test, and such progress was greatly reversed after co-treatment with the PPARγ antagonist, GW9662. TEL treatment also amended the cognitive deterioration through a PPARγ action, but to a less extent than PIO. Previous studies reported that TEL could inhibit amyloid β (Aβ)-[30, 31], streptozotocin-[37], and cerebral ischemia-induced [35] cognitive decline partly through PPARγ action, which agrees with our finding. PIO was reported to increase the cognitive capacity in animal models of PD [56] and AD [57]. Similarly, rosiglitazone, another PPARγ agonist, was reported to enhance cognitive function in diabetic mice [58].

The present data revealed that 3-NP prompted a significant fall in the final body weight. Typically, HD is often accompanied by considerable unintended weight loss that is probably an artifact of a hypermetabolic state and interference with cellular energy production [59]. In addition, the decreased motor ability may be partly responsible for reduced appetite and food intake in the 3-NP-treated animals [2]. As observed, TEL treatment did not show any further change in body weight, compared to the 3-NP group. This observation relies on the fact that TEL promotes an increase in caloric expenditure and mitochondrial energy metabolism, as well as decreases adipocyte size and accumulation in skeletal muscle and thus protects against weight gain [60]. This weight loss may be observed after giving high dosages of the drug and tend to be a class effect and independent of PPARγ activation [61, 62]. In contrast, PIO exhibited a positive effect on body weight, and this finding is in agreement with prior studies in which PIO reversed weight loss associated with chronic stress [63] as well as quinolinic acid [19] and MPTP intoxications [50]. Weight gain occurring with PIO may be attributed to its adipogenic and lipogenic characteristics [64], and a tendency for fluid accumulation due to renal sodium retention [65], which are all dependent on its PPARγ action.

Emerging evidence implicates the activation of microglial cells as a crucial event in the pathogenesis of HD [66]. Most of the anti-inflammatory action of PPARγ and their ligands stems from their PPARγ activation in glial cells [11]. Reactive microglia release several cytotoxic pro-inflammatory cytokines including TNF-α, IL-1β, and IL-6 [10]. Such pro-inflammatory molecules have been early reported to be repressed upon activation of PPARγ in monocytes and monocyte-derived macrophages [67]. In addition, PPARγ activation can regulate inflammatory pathways by transrepression of the transcription factor NF-κB [12], which is critical for HD disease progression [68].

Iba-1 is a selective microglial marker protein that reflects active microglial cells [69]. Our results indicated that 3-NP dramatically elevated the expression of Iba-1^+^ microglia and alters the expression/release of the pro-inflammatory factors, TNF-α, IL-1β, IL-6, and NF-κB, which is corroborated by several studies [70–73]. After treatment with PIO or TEL, we detected the repression of these inflammatory factors as a result of inhibition of both striatal microglial cells and NF-κB expression. Such anti-inflammatory effects were greatly abolished by concurrent administration of GW9662.

The current results agree with previous findings that showed that PPARγ activation downregulates brain inflammation by inhibiting several functions accompanying microglial activation [11], and protected against cerebral ischemic damage [74] and dopaminergic cell death [75–78]. **Sauerbeck et al. **[7] showed opposite results, reporting that PIO inhibited cortical microglia in a receptor-independent way in an animal model of traumatic brain injury (TBI).

In line with our findings, TEL protected against neuroinflammation through PPARγ-mediated inhibition of microglia in PD [34], cerebral ischemia [33], and TBI [79] animal models. Notably, candesartan, an ARB that exhibits less PPARγ activity than TEL, was reported to decrease the number of Iba-1^+^ cells after TBI dependently on PPARγ activation [80]. In addition, previous in vitro studies [26, 27, 29, 81, 82] conducted on human monocytes, cultured microglia, neuroblasts, and primary neurons reported that the direct anti-inflammatory protective effects of TEL may be attributed not only to its AT1R blockade characteristics but also to the PPARγ stimulation. Moreover, TEL effectively attenuated the increase in Aβ-induced expression of cytokines in animal models of AD [31, 83], where these anti-inflammatory effects were reversed by the PPARγ antagonist, GW9662. Besides, TEL suppressed microglial activation and inflammatory cytokines release in animal models of cerebral ischemia [84] and AD [85]. All of these findings support the idea that PPARγ activity of TEL strongly contributed to its ability to dampen microglial activation and hence control the neuroinflammatory state.

IL-10, a cytokine with anti-inflammatory functions, confines the inflammatory processes by suppressing NF-κB activity and proinflammatory cytokines production [86]. Besides the inhibition of 3-NP-induced proinflammatory cytokines, TEL and PIO enhanced IL-10 expression and the PPARγ antagonist partially reversed this effect. **Wang et al. **[29] and **Xu et al. **[87] supported this finding and reported the upregulation of PPARγ-mediated IL-10 in cultured BV-2 cells in response to TEL treatment. As pointed out in rat models of cerebral ischemia, treatment with TEL significantly decreased the pro-inflammatory cytokine levels and elevated the anti-inflammatory IL-10 levels [55, 88].

Mitochondria engage in several biological cellular functions that control cellular apoptosis and ATP production. PGC-1α, the orchestrator of mitochondrial function, is found to be repressed in HD patient and mouse striata, and contribute to the mitochondrial dysfunction, behavioral phenotype, and pathogenesis of HD [89, 90]. PGC-1α binds to nuclear respiratory factors (NRF-1 and NRF-2), which regulate many nuclear-encoded mitochondrial genes, including cytochrome c, mitochondrial transcription factor A (TFAM), and the respiratory chain complexes, thereby influencing cellular energy production [16]. Earlier studies reported downregulation of PPARγ in the striatum and peripheral tissues of HD transgenic animals [14] and mutant huntingtin (mHTT)-expressing striatal cells [17], suggesting that PPARγ-activating drugs could protect striatal cells from the mHTT-induced energy deficit.

Our results reported reduced mRNA levels of PPARγ, PGC-1α, and its downstream mitochondrial gene TFAM, among 3-NP rats. On the other hand, treatment with TEL and PIO enhanced the expression of PPARγ, PGC-1α, and TFAM. Moreover, ATP levels and mitochondrial SDH activity were concurrently increased. PPARγ blockade had little or no effect on the ability of TEL to increase TFAM expression, ATP level, and SDH activity, signifying that TEL is affecting these mitochondrial parameters through pathways that do not depend on PPARγ activation. Prior in vitro studies found that TEL upregulates mitochondrial function in human coronary artery endothelial cells [91] and skeletal muscles [92] through activation of AMP-activated protein kinase, a known upstream of PPARγ. In contrast, PPARγ blockade significantly attenuated the ability of PIO to increase the mitochondrial parameters. In the quinolinic acid rat model of HD, PIO improved mitochondrial enzyme complexes, cellular oxidative defense, and enhanced motor function in a PPARγ-dependent manner [19]. Rosiglitazone preserve mRNA levels of PGC-1α and improve motor impairment in transgenic HD mice [18]. In human neural stem cells, rosiglitazone was salvaged from Aβ-induced mitochondrial dysfunction through PPARγ-dependent mRNA upregulation of PGC-1α and mitochondrial genes (NRF-1 and TFAM) [93].

Compelling evidence implicates GSK-3β in HD pathogenesis, and its inhibition is a great neuroprotective factor [94]. GSK-3β is constitutively present in an active form and becomes inhibited by its phosphorylation at Ser9. This inhibitory phosphorylation is driven by phosphorylated Akt/protein kinase B and hence suppresses GSK-3β ability to initiate an apoptotic pathway [22]. It is worth noting that GSK-3β activity was demonstrated to regulate PPARγ activity [95], and the two PPARγ agonists, PIO, and rosiglitazone have been reported to enhance hippocampal Akt/GSK3β phosphorylation in animal models of AD [57, 96]. In our study, 3-NP exposure diminished pAkt and pGSK-3β expressions, which coincides with a recent report [97]. This effect occurred concomitantly with an increase in caspase-3 protein content, which is the final mediator of apoptotic cell death. PIO and TEL enhanced pAkt and pGSK-3β expressions, thus inhibiting the GSK-3β pro-apoptotic function. In cultured primary neurons, TEL curbed the suppression of the Akt/GSK3β pathway and caspase-3 activation following glutamate injury [81] and nutrient deprivation [28], via PPARγ. More recently, Kwon et al. [98] and Rasheed and Ibrahim [99] have reported that the PI3K/Akt/GSK3β pathway mediated the neuroprotective effect of TEL through PPARγ, which are consistent with our findings. From these results, it is suggested that activating PI3K/Akt/GSK3β pathway by TEL plays a crucial role in enhancing cell survival.

Furthermore, TEL and PIO increased the expression of the neuroprotective Bcl-2 gene, which is implicated in HD [100]. This is of particular concern because Bcl-2 is a downstream target of PPARγ, evidenced by a partial attenuation of Bcl-2 expression by co-treatment with GW9662. This finding is in harmony with a previous study, which has shown that TEL increases Bcl-2 in a PPARγ-dependent manner [28]. In addition, Haraguchi et al. [32] and Eslami et al. [101] suggested that TEL controls neuronal apoptosis via a PPARγ-dependent inhibition of caspase-3. Napolitano et al. [20] provided evidence in a mouse model of HD that PIO ameliorated the 3-NP-induced decline of striatal Bcl-2. Similarly, rosiglitazone amended the formation of mHTT aggregates and the reduction of Bcl-2 in the striatum [14]. Moreover, this PPARγ agonist protected against N2-A cell cytotoxicity and Aβ-induced apoptosis through a PPARγ mechanism that involves increased expression of Bcl-2 [93, 102, 103]. Interestingly, Bcl-2 is another downstream target of Akt. Rosiglitazone enhanced Akt/Bcl-2 expression through PPARγ-mediated expression of the neuroprotective protein neurotrophic factor-α1 [104, 105]. On the contrary, Fuenzalida et al. [102] reported that PPARγ overexpression in hippocampal neurons increased Bcl-2 protein content independently of Akt signaling. These results indicate that TEL influences AKT/GSK3β, Bcl-2, and caspase-3 expression via PPARγ, and thus controls apoptosis. Notably, this prosurvival action was further confirmed by an observable enhanced histopathological picture and increased number of viable neurons, as shown by Nissl stain.

In this study, we targeted the PPARγ action of TEL rather than the AT1R blockade action. A study [99] has recently demonstrated a marked expression of AT1R after 3-NP intoxication. Nevertheless, the role of AT1R as well as the whole renin-angiotensin system components is still controversial in HD and needs to be further investigated [106]. However, our results do not exclude the participation of AT1R antagonism in mediating the neuroprotective effects of TEL in this model. Taking into consideration that the neuroprotective effect was demonstrated by both the PPARγ full agonist PIO and the PPARγ partial agonist TEL and that the neuroprotective effect of TEL was partially diminished by the PPARγ antagonist GW9662, we sought that the role of TEL as a PPARγ partial agonist may be greater than that of AT1 in mediating such neuroprotective effects. As outlined in our study, it can be concluded that PPARγ could be exploited to prevent and/or treat inflammatory and apoptotic conditions in neurodegenerative diseases including HD.

## Data Availability

The datasets generated and/or analyzed during the current study are available from the corresponding author upon reasonable request.

## Abbreviations

Akt: Protein kinase B
ARB: Angiotensin II type 1 receptor blocker
ATP: Adenosine triphosphate
Bcl-2: B cell lymphoma-2
GSK-3β: Glycogen synthase kinase-3β
HD: Huntington’s disease
Iba-1: Ionized calcium-binding adaptor molecule-1
mHTT: Mutated huntingtin protein
3-NP: 3-Nitropropionic acid
NF-κB: Nuclear factor kappa B
PGC-1α: PPARγ co-activator-1 alpha
PPARγ: Peroxisome proliferator-activated receptor-gamma
SDH: Succinate dehydrogenase
TFAM: Mitochondrial transcription factor A
